# Research on imaging method of driver's attention area based on deep neural network

**DOI:** 10.1038/s41598-022-20829-w

**Published:** 2022-09-30

**Authors:** Shuanfeng Zhao, Yao Li, Junjie Ma, Zhizhong Xing, Zenghui Tang, Shibo Zhu

**Affiliations:** School of Mechanical Engineering, Xi’an University of Science and Technology, Xi’an , 710054 China

**Keywords:** Electrical and electronic engineering, Optical sensors, Human behaviour

## Abstract

In the driving process, the driver's visual attention area is of great significance to the research of intelligent driving decision-making behavior and the dynamic research of driving behavior. Traditional driver intention recognition has problems such as large contact interference with wearing equipment, the high false detection rate for drivers wearing glasses and strong light, and unclear extraction of the field of view. We use the driver's field of vision image taken by the dash cam and the corresponding vehicle driving state data (steering wheel angle and vehicle speed). Combined with the interpretability method of the deep neural network, a method of imaging the driver's attention area is proposed. The basic idea of this method is to perform attention imaging analysis on the neural network virtual driver based on the vehicle driving state data, and then infer the visual attention area of the human driver. The results show that this method can realize the reverse reasoning of the driver's intention behavior during driving, image the driver's visual attention area, and provide a theoretical basis for the dynamic analysis of the driver's driving behavior and the further development of traffic safety analysis.

## Introduction

In the development of unmanned driving, the interpretable issues in the decision-making process of unmanned vehicles and the ethical issues involved are obstacles to the promotion and application of unmanned driving. The interpretability study of the intermediate process of unmanned vehicles from scanning to the external driving environment to execution of actions, especially the mapping relationship between the driving behavior of unmanned vehicles and the driving behavior of human drivers, is an urgent need to be solved. The driver's visual attention area during driving is the core issue in our dynamic driving behavior research. The driver's attention detection was carried out in the past study through methods such as changes in ECG data, head posture, and gaze tracking. Using the driver's visual field image data and the corresponding vehicle driving state data (steering wheel angle and vehicle speed) to reverse the driving behavior habits during driving is the focus of our research. The wide application of neural networks in various fields provides support for our work^1,2^.

The need to explain self-driving behavior is multi-factorial. To begin with, autonomous driving is a high-stake and safetycritical application. It is thus natural to ask for performance guarantees, from a societal point-of-view. However, driving models are not completely testable under all scenarios as it is not possible to exhaustively list and evaluate every situation the model may possibly encounter. As a fallback solution, this motivates the need for explanation of driving decisions^3^. Nowadays, with the wide application of neural networks in various fields^4^, the transparency and interpretability of deep neural networks have attracted the attention of researchers. Essentially, the final extraction and presentation of the driver's attention region mainly relies on the interpretability approach of deep neural networks. Koo et al.^5^ shows that if the autonomous driving system can provide the user with the reasons for their decision-making behavior, the user's trust in the autonomous driving system can be significantly enhanced. Decrypting the intermediate process of the deep neural network and seeking the reliability of the output of the deep neural network not only improves the credibility of the engineering application of deep learning technology, but also makes the output of the neural network more acceptable to people^6^. As Autonomous Vehicles rely more and more on deep neural networks processing visual streams it is of critical importance to study the explainability of driving models from a computer vision perspective^7^. Especially in the field of intelligent driving vehicles, it is bound to face a large number of ethical issues, and the reliability of the output results of the deep neural network and the interpretability of the learning process of the deep neural network provide technical support for this problem. Interpretability not only enhances the driver's trust in the autonomous driving system, but also helps the developers of the autonomous driving system to effectively design, debug and diagnose the autonomous driving system. Explainability also makes autonomous driving behaviour predictable, verifiable, and auditable. For governments and policymakers, explainability can also drive compliance for autonomous driving systems.

In the research of driver's driving behavior, especially the driver's visual attention, some previous work conducted attention analysis based on the driver's physiological characteristic data. Research shows that whether the driver’s attention is concentrated, the changes in the ECG signal under different eye movements are different. when the driver fatigue distraction, ECG decreased significantly^8^.

Used EEG acquisition equipment to collect drivers' EEG signals and detect fatigued driving and distracted driving^9,10^. Wu et al.^11^ used the ECG signal to detect the driver's attention. Driving behaviour analysis based on physiological characteristics has made significant achievements in studying driver behaviour and has high reliability under experimental conditions. However, the physiological characteristic data collection device needs to be worn on the head or chest and abdomen in contact, which restricts the driver's operation, interferes with the driver's normal driving behaviour, and cannot reflect the driver's attention in a natural traffic environment. Our method is based on a deep neural network to image the driver's attention area to realize the evaluation and detection of the driver's attention.

With the continuous progress and development of computer vision technology, contact detection methods have been unable to meet the requirements of driver attention area extraction, and non-contact detection has become a key technology for driver attention prediction. Drivers no longer wear data collection equipment, but rely on cameras to capture the driver's facial features (eye movement status, fatigue level) and the driver's head posture to predict the driver's attention. Researchers have conducted related research on non-contact detection methods: Choi et al.^12^. By tracking the driver's pupil, reflecting the driver's line of sight according to the pupil position, estimating the driver's attention direction, avoiding wearing the device in terms of judging the driver's attention, Chutroian also implemented head pose-based driver attention detection in their work^13^. In studies^14–17^, the driver's attention was judged by detecting the driver's eyes and facial features. Sigari et al.^18^ captured the driver's face and eye information to determine the driver's attention state, which played an important role in vehicle active safety. To further analyze the accuracy of the driver's visual attention direction, Morando et al.^19^ systematically analyzed the driver's eye tracking data in 2019 and concluded that the driver's visual response is inseparable from the driving environment. Lee et al.^20^ proposed an eye-tracking system that requires the experimenter to watch a large display at close range, under the premise that the gaze point can be estimated with high accuracy, however, this method is not suitable for Hardware and experimental environment requirements are high. Similarly, eye movement measurement equipment has been used to obtain the driver's visual attention distribution^21^ to determine the driver's visual attention in a specific situation at an intersection, which has contributed to the study of the driver's attention area. Tests have been carried out on straight and turning roads according to actual driving conditions^22^. The use of head-mounted eye trackers to collect driver's eye movement data can stably and accurately track the driver's eyeball attention area. However, the direct contact of the head-mounted eye tracker or contact device with the driver will cause interference to the driver's head, eyes, and neck movement to a large extent. There is a difference between the driver's psychological state and the actual driving state, which ultimately affects the driver's attention area collection and imaging in the actual situation.

The above situation is carried out under specific interference, and the details of the captured attention area are not precise. We propose a driver's visual field image data and vehicle driving state data (steering wheel angle and vehicle speed) of the driver that the driving recorder can capture to reverse the driver's visual attention area through the imaging method. This method avoids the interference caused by the wearable detection device to the normal driving of the driver, and at the same time, visualizes the attention area of the attention. Our work contributions are as follows:

1. Based on the deep neural network, the driver's attention area imaging is proposed. The correlation feature is extracted from the vehicle driving state data and the driver's field of view image data. The direct influence of interfering equipment on the extraction of the driver's attention area is avoided. Of course, the conditions for implementing our method are more relaxed.
2. A deep neural network imaging method is proposed to display the critical areas of the driver's visual attention. It straightforwardly show a particular area of concern and a specific target. The direct relationship of the attention area is determined by combining the vehicle driving state data.
3. According to the size of the network attention weight, the attention area is allocated in the image. Further, exposing the intermediate process of the neural network. Enhance the interpretability of the network model.

**Figure 1:**
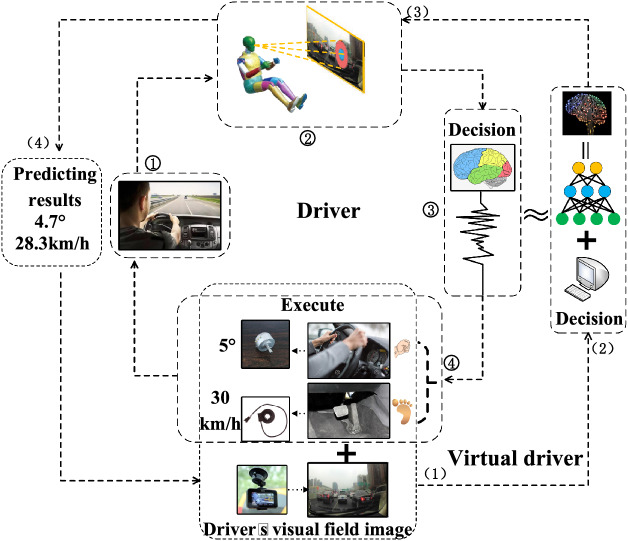
Comparison of the driving process of a human driver and a virtual driver.

As shown in Fig. 1, there are two closed loop dashed paths from the inside to the outside. The dotted line in the inner circle of the transmission path represents the driving reaction process of the human driver. First, the road conditions are observed (➀), and the visual attention area information (➁) is input into the brain. The brain makes decisions (➂) and controls the limbs to change the steering wheel and vehicle speed (➃). Our model is regarded as a virtual driver, and the dotted line in the outer circle is the backtracking process of the virtual driver. The virtual driver is a human driver's brain twin system (2) built using deep neural networks. It takes the driver's visual field image data and the corresponding vehicle driving state data (steering wheel angle and vehicle speed) as input (1) for training. The steering wheel angle makes predictions with vehicle speed (4) and outputs the same execution result as a human driver. In the end, it is deduced which area the driver pays attention when he manipulates the vehicle to accelerate, decelerate, and turn to perform actions (3). Our idea is to turn the driver's attention imaging method into a deep neural network visualization problem to infer the human driver's visual attention area.

## Related works

**Figure 2:**
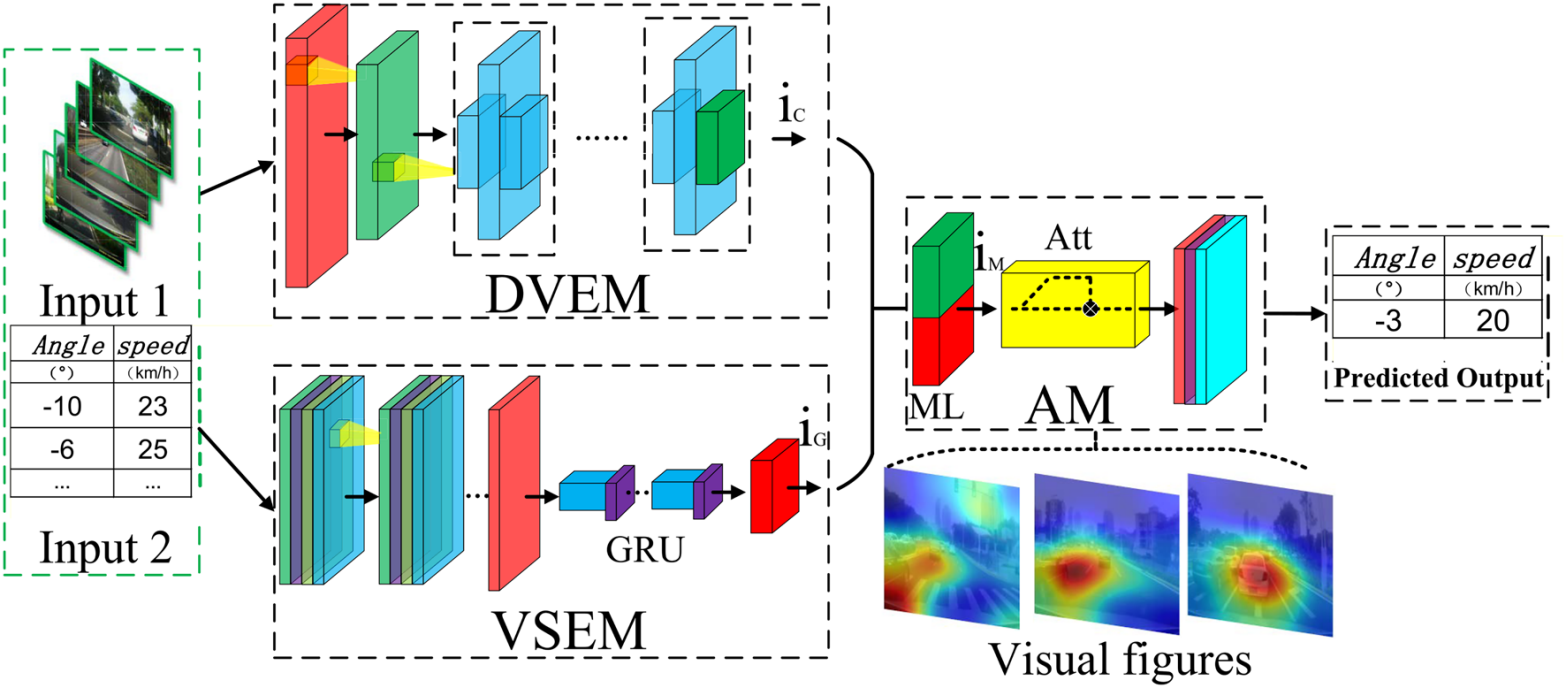
The structure diagram of the driver's attention imaging method.

According to the driver's field of vision image data and vehicle driving state data (steering wheel angle and vehicle speed), we propose a network model as shown in Fig. 2, which includes driver field of vision information extraction module (DVEM), vehicle driving state data extraction module (VSEM), attention module (AM). The network model is trained through a large number of images corresponding to continuous driver visual field data and vehicle driving state data (steering wheel Angle and vehicle speed), and the real perception method of deep neural network is extracted, and then attention imaging is performed.

### Driver field of vision information extraction module (DVEM)

**Figure 3:**
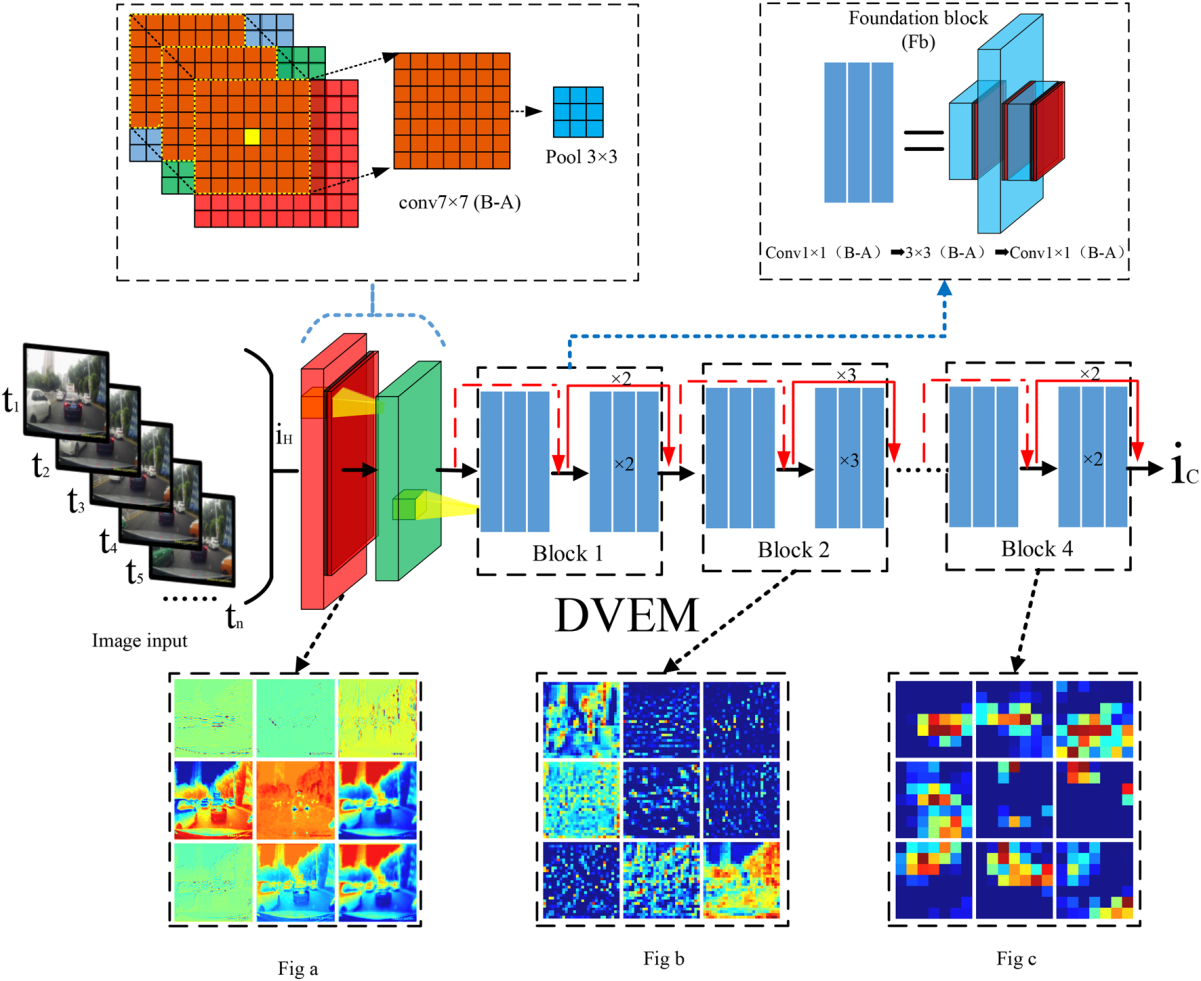
The structure of the driver field of vision information extraction module (DVEM).

In the actual scene of the vehicle driving, the driver captures a variety of external information. The influence of visual information on the driver's control of the vehicle is significant. The driver's eyes' perception of the external environment and the scene presented in the brain is similar to the continuous images captured by the camera. We use convolutional neural networks to extract features from the driving recorder images collected when driving. In our method, the driver field of vision information extraction module (DVEM) extracts features from the driver's visual image. The DVEM network structure is shown in Fig. 3. The proposed residual structure^23^ dramatically improves the feature extraction performance of deep convolutional neural networks, and it is widely used in the field of vision^24^. The image passes the 7 × 7 convolution with a step size of 2, the output channel of the convolution layer is 64, and the batch normalization layer^25^ and the ReLU activation layer^26^, and the 3 × 3 Maxpooling completes the preliminary information extraction. While ensuring the completeness of the image information, the amount of parameters is reduced.

In the model structure of DVEM, 1 × 1 convolution, 3 × 3 convolution, and 1 × 1 convolution are combined with the batch normalization layer and the activation layer to form a Foundation block (Fb). This Fb residual combination constitutes Block i with more layers (i = 1, 2, 3, 4). Block 1 and Block 4 are composed of 3 Fb, and Block 2 and Block 3 contain 4 and 9 Fb respectively.

### Vehicle driving state data extraction module (VSEM)

**Figure 4:**
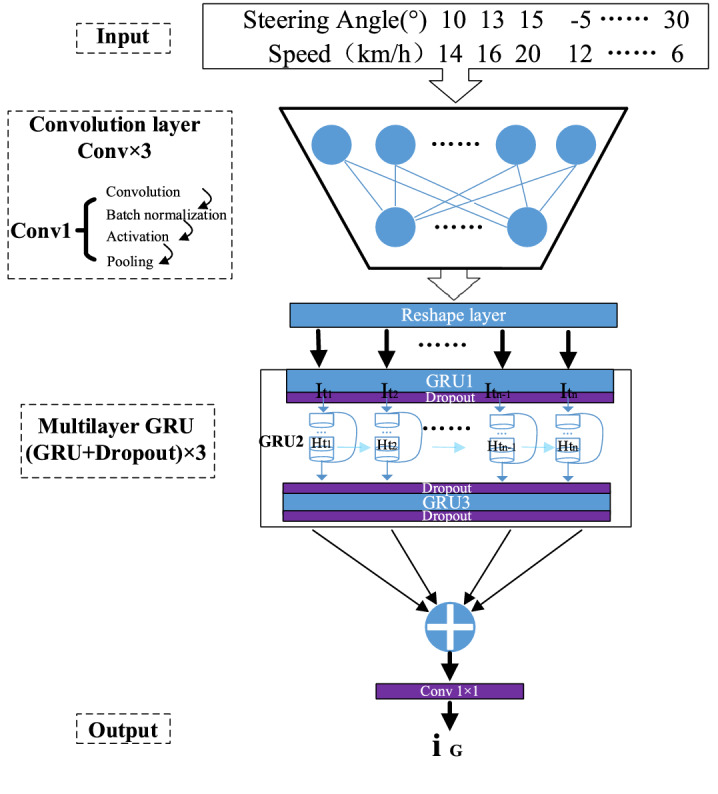
The structure of the vehicle driving state data extraction module (VSEM).

VSEM takes the past, and continuous vehicle driving state data as input for feature extraction and finally realizes vehicle driving state data prediction. The past continuous vehicle driving state data is time series data. The optimized networks Long Short Term Memory (LSTM)^27^ and Gated Recurrent Unit (GRU)^28^ derived from the continuous development of Recurrent Neural Network (RNN) can effectively avoid gradient disappearance and explosion when processing time series data. In our VSEM, the GRU module is introduced to capture the relevant information between the long and short term data in the past vehicle driving state data and to extract information for the generation of prediction results. Figure 4 shows the VSEM structure information, input vehicle driving state data, after Conv1, Conv2, and Conv3, after a total of 3 layers of convolution, perform a Reshape operation on the data to prepare for the subsequent input to the GRU layer. Multi-layer GRU is the vital part of the entire module to ensure time series information. We have constructed a three-layer GRU for feature extraction of vehicle driving state data. In order to prevent overfitting, the Dropout^29^ layer is added after each layer of GRU to reduce the model's excessive dependence on training data and improve the generalization ability of the model.

**Figure 5:**
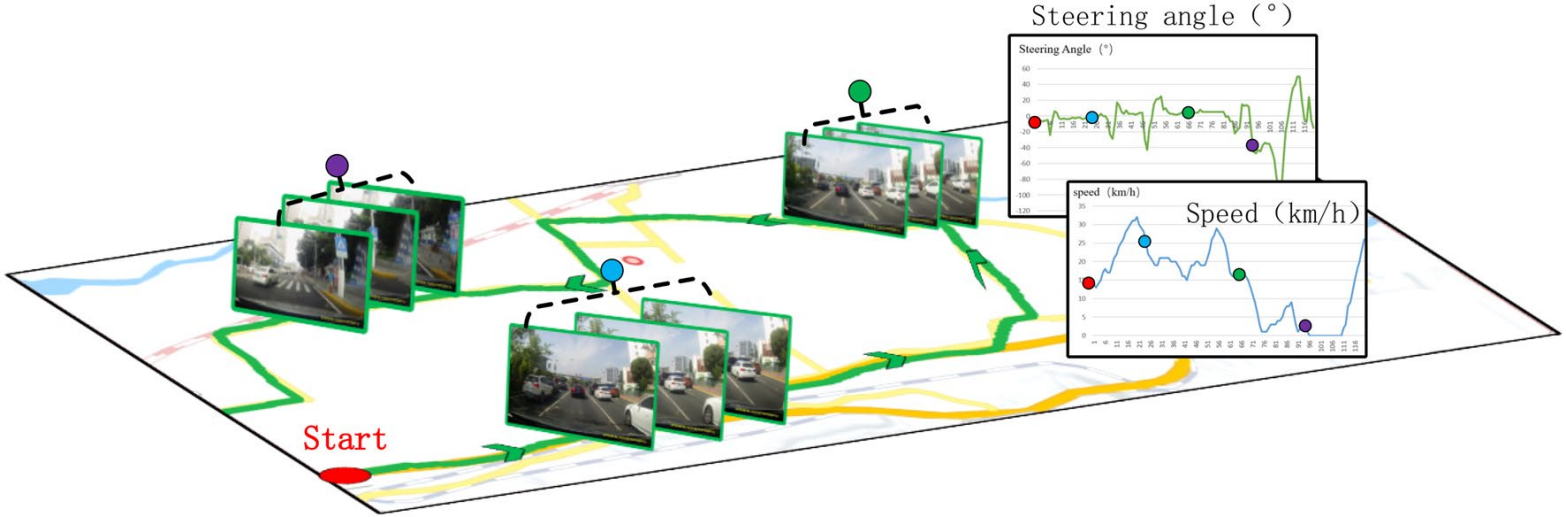
Dataset (contains continuous image data and its corresponding continuous vehicle driving state data).

$\widetilde{{I_{t} }}$ is the current information. The memory information $H_{t}$ at each moment depends on the memory information $H_{t - 1}$ saved at the previous moment and the current information $\widetilde{{I_{t} }}$. The uniqueness of GRU is that it is optimized on the basis of the LSTM gating structure. It includes two gating units of reset gate $R_{t}$ and update gate $U_{t}$. The input of reset gate and update gate are the same, respectively $H_{t - 1}$ and $I_{t}$. The role of update gate in GRU is equivalent to the combination of input gate and forget gate in LSTM. $1 - U_{t}$ can be regarded as input, and $U_{t}$ can be regarded as the part that is randomly forgotten. Figure 5 shows the gating structure of the GRU, where the green part represents the reset gate and the red part represents the update gate. The generation of $\widetilde{{I_{t} }}$ is closely related to $H_{t - 1}$ and reset gate $R_{t}$. In the calculation of $\widetilde{{I_{t} }}$, ${\text{f}} = \tanh \left( x \right) = \frac{{e^{x} - e^{ - x} }}{{e^{x} + e^{ - x} }}$.

$R_{t}$ and $U_{t}$ are processed by the sigmoid = λ(x) activation function, and their values are controlled between 0 and 1, which is the same as the fully connected layer here. Where $\omega$ and $b$ represent the weight matrix and the bias matrix respectively.

1 $$ H_{t} = \left( {1 - U_{t} } \right)*H_{t - 1} + U_{t} *\widetilde{{I_{t} }} $$

2 $$ \widetilde{{I_{t} }} = {\text{f}}\left( {\omega_{a} \left[ {R_{t} *H_{t - 1} ,I_{t} } \right]} \right) + b_{a} $$

3 $$ U_{t} = {\uplambda }\left( {\omega_{b} \left[ {H_{t - 1} ,I_{t} } \right] + b_{b} } \right) $$

4 $$ R_{t} = {\uplambda }\left( {\omega_{c} \left[ {H_{t - 1} ,I_{t} } \right] + b_{c} } \right) $$

### Attention module (AM)

The attention mechanism^30,31^ generates attention perception features (emphasis features) in the input information according to different weight parameters, highlights important information while suppressing irrelevant and unimportant information. The application of attention mechanism in neural network improves the interpretability of network attention information. After the image input is extracted by DVEM, the mapping information ${\text{i}}_{C}$ is obtained, and the vehicle driving state data is extracted by VSEM to obtain the mapping information ${\text{i}}_{G}$, which is merged in the attention module. In our method, the last layer of convolution of DVEM and VSEM is set to 1 × 1, and the purpose is to map the features extracted from the two parts to a common matrix, so that ${\text{i}}_{C}$ and ${\text{i}}_{G}$ can be used in the mixing layer (ML) in AM perform integration, and perform subsequent batch normalization and tanh activation operations in ML.

In AM, the attention mechanism is introduced after ML. The attention mechanism can directly focus on the part of the image and vehicle driving state data that has a positive effect on prediction. We use ${\updelta }_{A} \left( {P,D} \right)$ to represent the attention function, where P and D represent the image features and vehicle driving state data features respectively, and the partial weight vector θ of attention is calculated as:

5 $$ \theta_{i} = \frac{{exp\left( {\delta_{A} \left( {P_{i} ,D_{i} } \right)} \right)}}{{\mathop \sum \nolimits_{i} exp\left( {\delta_{A} \left( {P_{i} ,D_{i} } \right)} \right)}} $$

6 $$ {{ \delta }}_{A} \left( {P,D} \right) = {\upomega }\left[ {C_{P \to D} ;C_{D \to P} } \right] + {\text{b}} $$

7 $$ C_{P \to D} = {\text{tanh}}\left( {\omega_{D} D + \left( {\omega_{{P^{\prime}}} \Delta \left( P \right)} \right)} \right) $$

8 $$ C_{D \to P} = {\text{tanh}}\left( {\omega_{P} P + \left( {\omega_{{D^{\prime}}} \Delta \left( D \right)} \right)} \right) $$

Among them, $\omega_{P}$, $\omega_{D}$, $\omega_{{P^{\prime}}}$, $\omega_{{D^{\prime}}}$ are the weight coefficients that change continuously with training, and b is the bias coefficient. In AM, the image and vehicle driving state data influence each other in the calculation of attention emphasis. The attention of the two is determined by their respective global vectors ∆(P) and ∆(D), $C_{P \to D}$ and $C_{D \to P}$ represents the interaction of two feature information. The final output layer is also included in the AM, through the global average pooling layer (GAP), fully connected layer and softmax activation output predicted steering wheel angle and vehicle speed results. GAP greatly reduces the amount of network calculation parameters, effectively reduces the dependence of the network model on training data, and realizes the conversion of feature vectors. We finally interpolate the visualized attention map to the size of the original image, overlay the original image, and merge the output as the visualized result of the driver's attention area.

## Experiment

### Dataset

In our method, the vehicle driving state data is key for us to use the model to find the driver's attention area. We use the DBNet published by the team of Professor Junli of Xiamen University and Professor Cewu Lu of Shanghai Jiao Tong University in 2018. Dataset^32^. DBNet contains the driving images we need from the driver's perspective and collects more than 100 kms of driving distance data, including urban roads, intersections, turning intersections, mountain roads, etc. At the same time, the dataset also contains the vehicle driving state data, that is, the steering wheel angle and vehicle speed information. The steering wheel angle is relative to the pre-specified standard 0°; the left turn is recorded as "−", and the right turn is recorded as " + ". Based on the DBNet dataset, we resize 1920 × 1080 images as our data for training, verification and testing. We choose the data format to display in Fig. 5. In the real driving environment, the driving recorder is used to collect image data, and the vehicle driving state data (steering angle (°) and speed (km/h)) is collected at the same time.

### Training

In our training, the hardware is a computer equipped with an NVIDIA GTX1080 graphics card, which is carried out under the Ubuntu 16.04 LTS operating system. The deep learning framework is PyTorch^33^, and the version of torch is 0.4.1. CUDA9.0 and cuDNN7.0 are also used in the operating system to accelerate training, support GPU calls, and accelerate the training process. Some parameter settings in training are given in Table 1.

**Table 1:** Training parameter settings.

Name	Value
learning rate	0.002
Batch size	12
Epoch	200
Decay	0.001
Dropout rate	0.2

**Figure 6:**
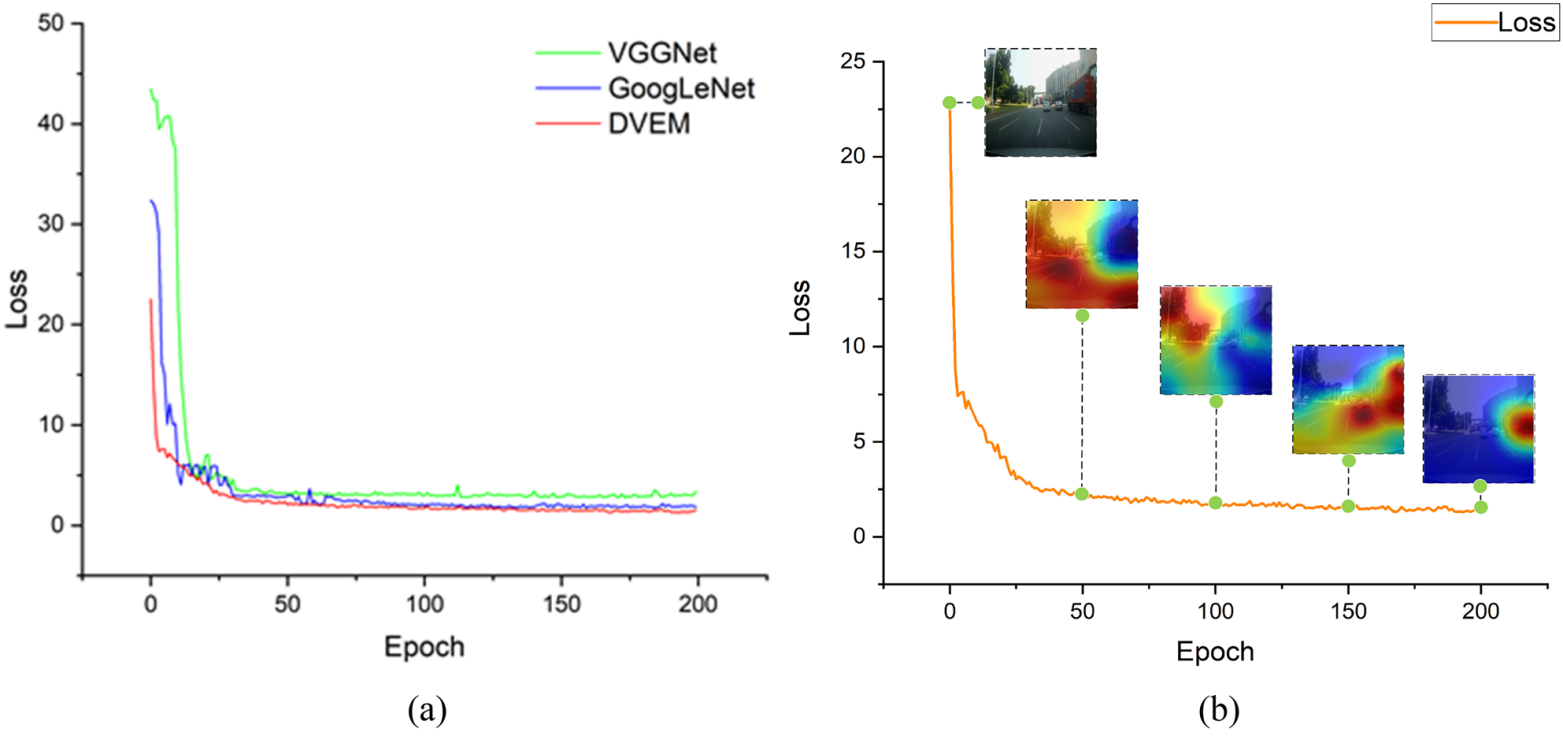
Training loss graph (**a**) DVEM training comparison with other models; (**b**) attention change graph during DVEM training.

For the parameter optimization problem of the data in the model, we chose the Adam optimizer (adaptive moment estimation)^34^, the Adam optimizer combines the traditional first-order optimization algorithm and the second-order optimization algorithm. Adam can optimize and modify the training parameters according to the continuous training of the network to speed up the convergence of the model. At the same time, the mean square error (MSE) is selected as the loss function. This is because our prediction of vehicle driving state data is for the regression task to generate specific Steering Angle (°) and Speed (km/h) values. In the formula, x and y represent the Steering Angle and Speed respectively, $\delta_{i}$ represents the true value, $\delta_{i}^{\prime }$ is the predicted value, and n is the number of data. Figure 6 shows the change of the training process. It can be seen that the loss value of the model decreases rapidly in the initial stage during the training process, and stabilizes after 50 Epochs, then the model finally successfully converges.

9 $$ L_{OSS} = MSE_{{\left( {\delta = x,\delta = y} \right)}} = \frac{{\mathop \sum \nolimits_{i = 1}^{n} \left( {\delta_{i} - \delta_{i}^{^{\prime}} } \right)^{2} }}{n} $$

Figure 6a is the training results of VGGNet16^35^, GoogLeNet^36^ and DVEM. The final training loss values of VGGNet16, GoogLeNet and DVEM are 3.37, 1.86 and 1.55, respectively. It can be seen in Fig. 6b that as the training Epoch continues to increase, the attention area is constantly changing. We will show the driver's attention area extraction effect at different Epoch (50, 100, 150, 200). It can be seen that the attention area gradually becomes smaller and more precise.

VSEM extracts the driver's field of view information, adds a multi-layer GRU structure to the model to extract the time series features in the image and vehicle field of view extraction information (steering wheel angle and vehicle speed), and introduces an attention module to enhance the model's ability to extract strong features. In order to verify the prediction accuracy of the VSEM model and the necessity of each model, the long-term and short-term memory network, the single-layer GRU prediction model, and the non-attention module model were used to predict the extraction information (steering wheel angle and speed) of the vehicle field of view. The results of the VSEM prediction model in this paper are compared.

**Figure 7:**
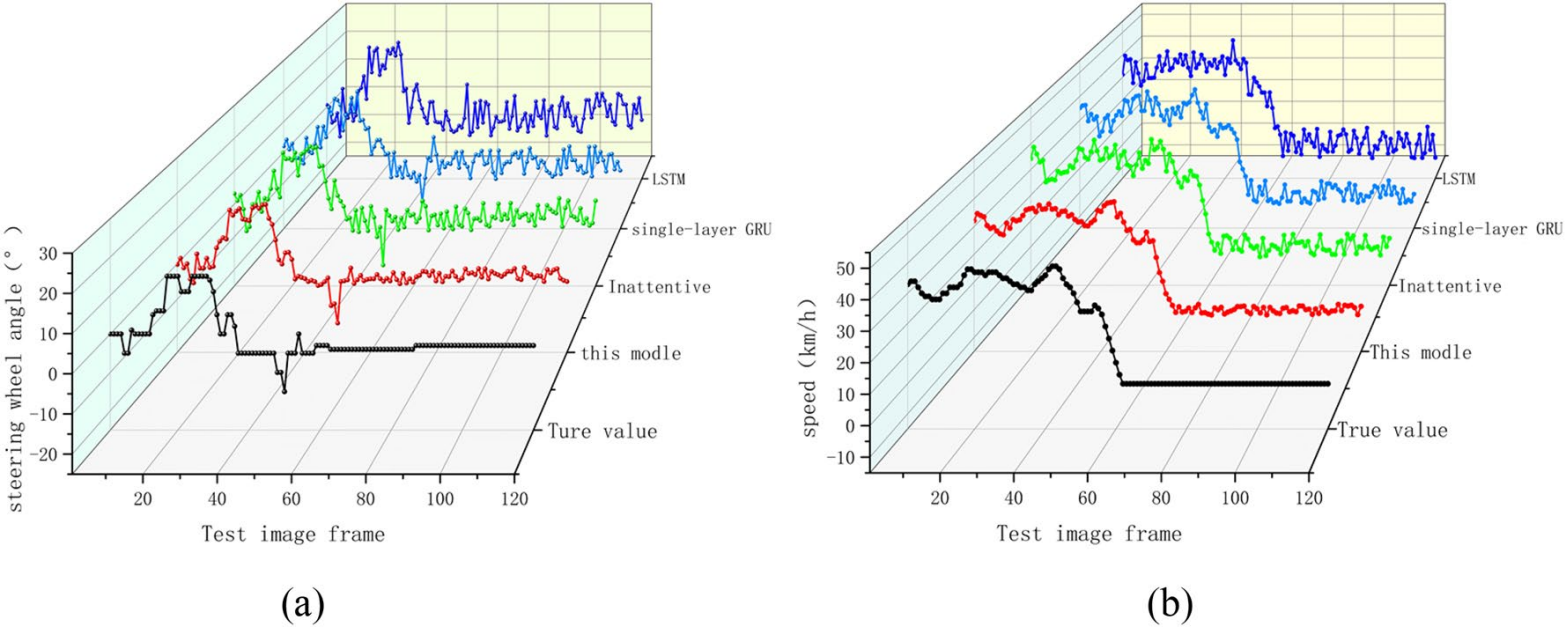
Comparison of vehicle driving data prediction results (**a**) steering wheel angle prediction; (**b**) predicted vehicle speed.

As shown in the figure is the comparison of the prediction results of VSEM and each model. Figure 7a is the prediction of the steering wheel angle by different models. Figure 7b is the prediction of the vehicle speed by different models. The black dotted line in the figure is real. Steering wheel angle data and vehicle speed data, the figure can intuitively see that in the two data predictions, the driving data prediction result of VSEM is a red curve. Compared with the prediction results of the other three models, the oscillation amplitude of the red curve is smaller and more close to the real data. At the same time, compared with the prediction results without attention, it is enough to prove that the introduction of the attention module improves the prediction accuracy of the data. The application of three-layer GRU makes the data prediction accuracy higher than that of single-layer GRU. The prediction data and errors of each model are summarized in Table 2.

**Table 2:** Comparison of prediction results of different prediction models.

FPS	Unit	1	.…	39	40	.…	80	81	…	120	MSE
True value	Angle	0.000	.…	− 5.000	− 5.000	.…	− 4.000	− 4.000	…	− 3.000	0.000
	Speed	33.000		35.000	36.000		0.000	0.000		0.000	0.000
This modle	Angle	− 0.213		− 3.872	− 4.187		− 4.843	− 2.668		− 4.986	0.362
	Speed	32.097		35.792	35.449		− 0.390	0.120		1.248	0.224
Inattentive	Angle	3.150		− 4.218	− 8.068		− 0.468	− 4.368		1.024	0.568
	Speed	34.229		33.603	32.333		− 1.470	0.656		3.293	0.623
Single-layer GRU	Angle	1.946		− 8.173	− 2.157		− 3.092	− 3.231		− 5.953	2.543
	Speed	35.111		36.662	37.532		− 3.345	− 2.654		− 0.783	2.896
LSTM	Angle	0.794		− 5.626	− 0.078		2.017	− 2.977		− 4.455	5.426
	Speed	31.183		39.346	31.105		5.698	− 3.068		− 5.794	5.867

**Figure 8:**
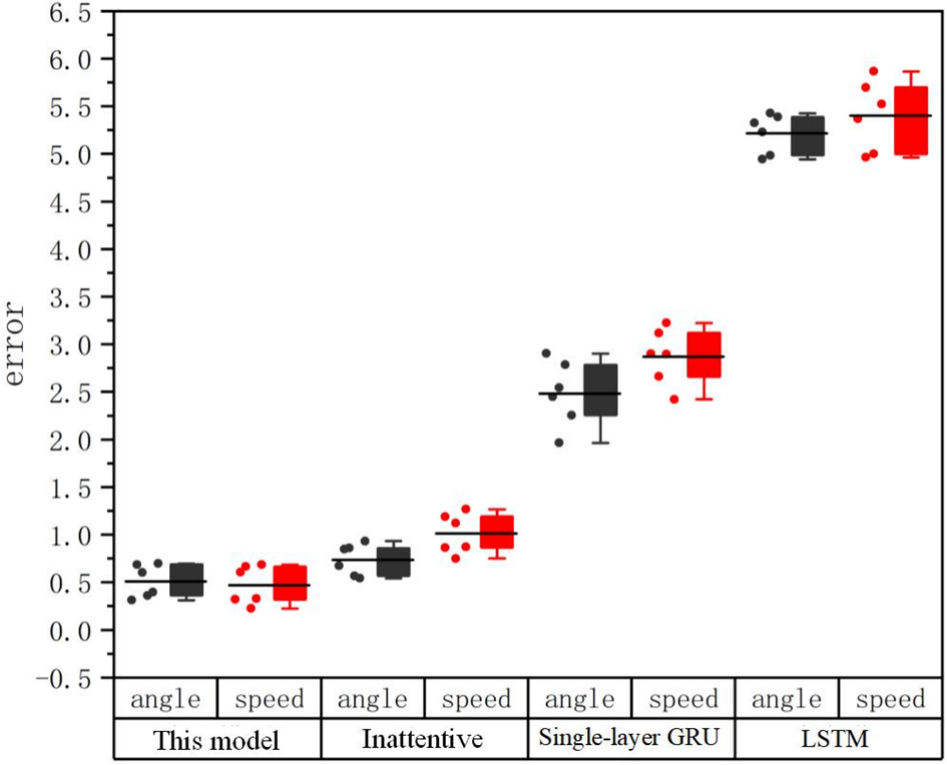
Comparison of mean square errors of predicted values of different models.

Using each prediction model, predict the continuous 6-segment vehicle driving data in the data set, and compare their mean square errors. Figure 8 shows the comparison chart of the mean square errors of the predicted values of each model.

The black box in Fig. 8 represents the mean square error set of the steering wheel angle prediction values of the four prediction models, the red box is the mean square error set of the vehicle speed prediction value, and the left point set of each box is the prediction results of 6 groups of different data The mean square error value, the black horizontal line between the point set and the box represents the mean square error average. As can be seen from the figure, from the comparison of the distribution of the mean square error of the predicted value, it is found that the VSEM model designed in this paper has the lowest mean square error among the four models in the prediction of steering wheel angle and vehicle speed, and also represents the prediction model of this paper. The predicted results are closest to the real data.

## Results and analysis

**Figure 9:**
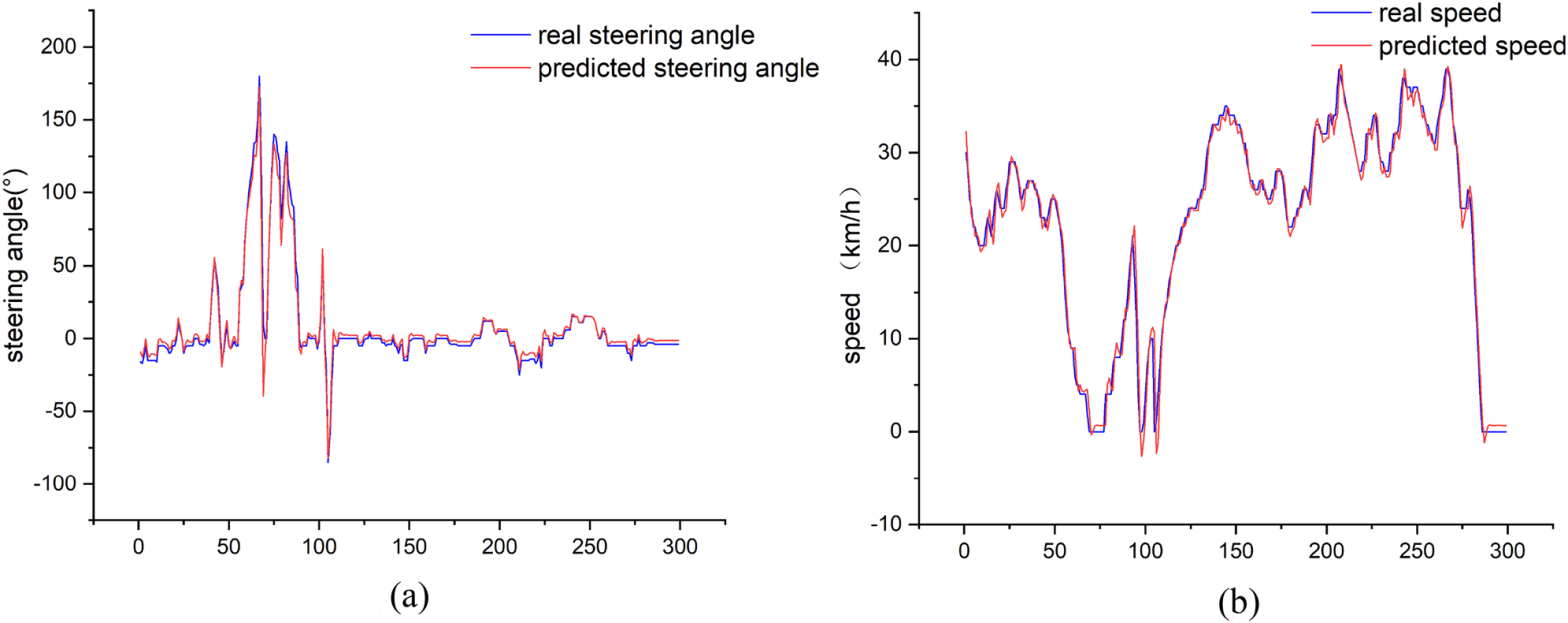
Steering wheel angle and vehicle speed prediction results.

VSEM makes predictions based on continuous steering wheel angles and vehicle speeds. Figure 9 shows the predicted values of the steering wheel angle and vehicle speed and compares them with the actual values. The predicted data and the real data have similar trends. According to the true value of the steering wheel angle and the predicted value, the average absolute error is 6.62. Similarly, the average absolute error of vehicle speed is 0.97.

**Figure 10:**
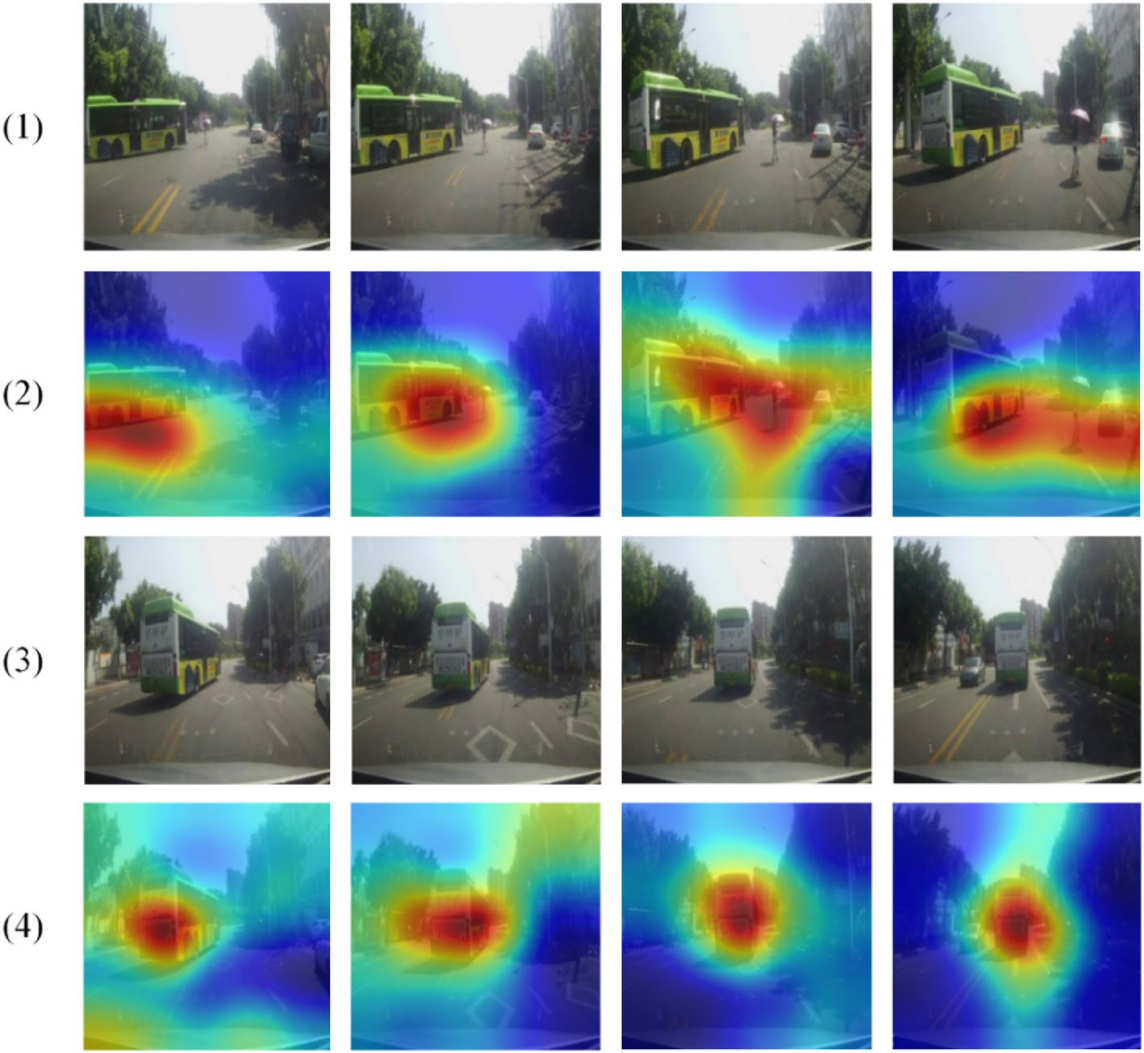
Imaging of driver's attention area.

The driver's attention area is shown in Fig. 10. Here, we select continuous test images for display and visualize the continuous images to show the dynamic driver's attention area change process. In Fig. 10, the images in lines (1) and (3) are the original images of the input model. The eight images in these two lines represent continuous driving scenes. The images in lines (2) and (4) are the attention map overlaying the original image. The colour of the attention area transitions from red to blue, where red represents the area with the most significant attention weight, that is, the key area of attention.

We can see that in the traffic scene shown in Fig. 10, a turning bus is encountered on the left front, and pedestrians appear in the middle of the road. According to the attention map we output, it is obvious that when turning to the bus, the key area to pay attention to is at the rear of the bus. As the vehicle moves, the distance to the pedestrian in the middle of the road gets closer and closer. The attention area starts from the tail of the bus and extends to the pedestrian on the right, and finally covers the part of the person in the area the image. The traffic scene shown in the image in line (3) is simpler than that in line (1). At this time, the pedestrian has left the camera collection area, and only one bus in the image. Therefore, the attention map in line (4) compared with line (2) has a single attention target, a smaller and more precise attention area. These signs are consistent with the driver's visual information capture.

**Figure 11:**
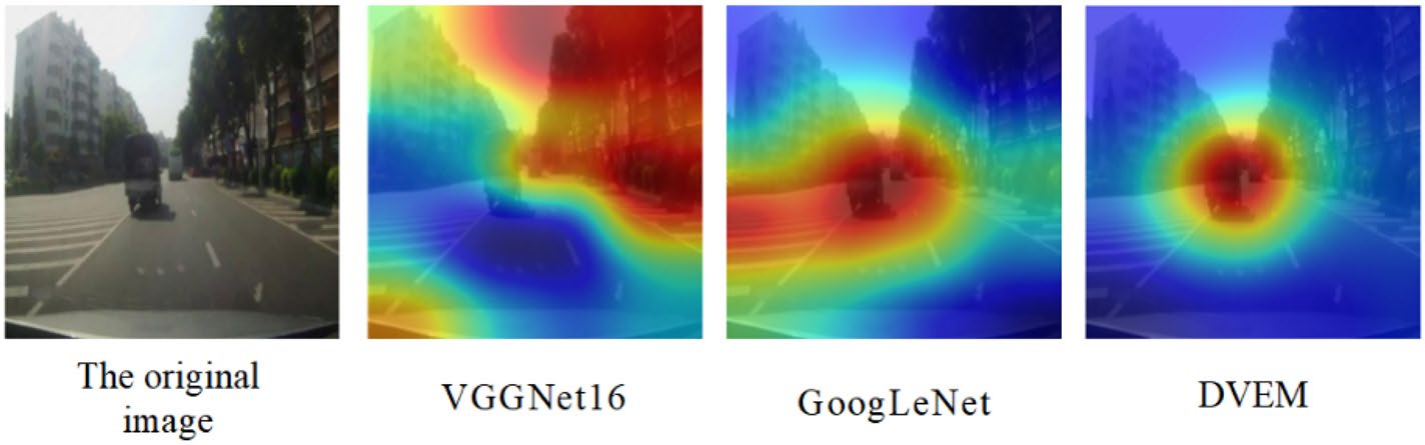
VGGNet16, GoogLeNet, DVEM imaging results.

In our method, DVEM mainly performs feature extraction for image information, which is a very important link. Here we use the classic VGGNet16, GoogLeNet model to replace DVEM, and the output result is shown in Fig. 11. After comparison, it is found that the final attention region extraction effect generated by the three models shows that the attention region visualization image generated by VGGNet16 almost occupies the upper right corner of the entire image. However, we observe the original image and find that the upper right corner is the street-facing building, which has less impact on the driver's operation than the truck in the middle of the road. Compared with the results of DVEM, the results of GoogLeNet and DVEM both focus on the vehicle in front. Still, GoogLeNet also extracts the extra area on the left side of the image, so our model extraction effect is better than VGGNet16 and GoogLeNet.

**Figure 12:**
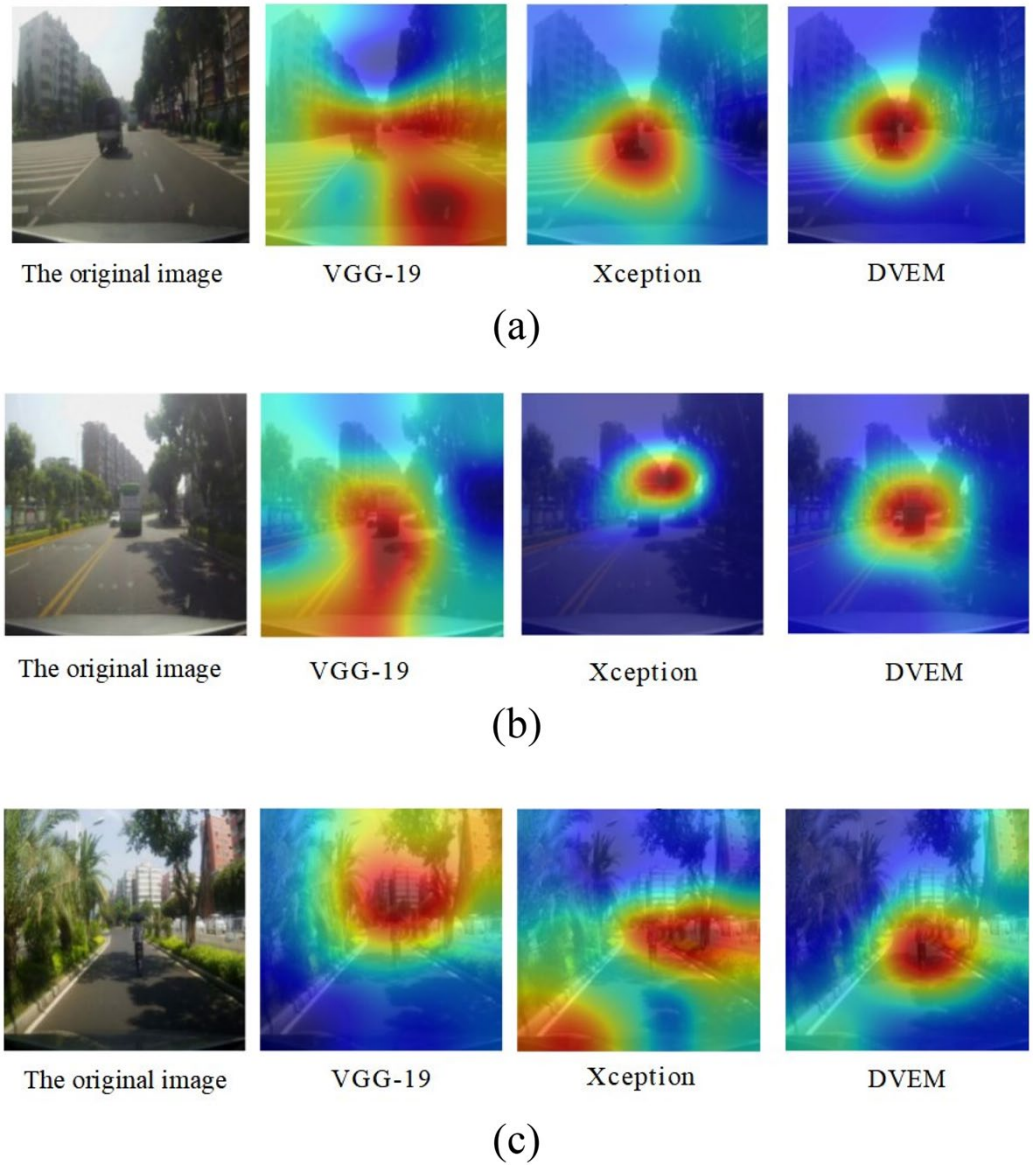
VGG-19, Xception, DVEM imaging results.

The comparison of the classic model is not enough to show the superiority of this model, so this paper uses VGG-19^37^, Xception^38^ and DVEM for comparative analysis, and the results are shown in Fig. 12:

Using VGG-19, Xception model to replace the DVEM in the driver's attention area extraction model in real traffic scenes, we compare through three different traffic scenes, and the final extraction results of the three models are shown in Fig. 12. In the analysis of Fig. 12a, in the attention area visualization map generated by VGG-19, the extracted attention area occupies almost the lower right corner of the whole picture. It is found that the lower right corner of the original map is a road, and there are no vehicles and pedestrians, which has little impact on the driver's operation. Compared with DVEM, Xception and DVEM both focus on the vehicle in front, but through the effect comparison, we can find that the effect of this model is better; In Fig. 12b, although VGG-19 focuses on the front vehicle, it also extracts the redundant area at the bottom left of the picture. Compared with the results of Xception and DVEM, although Xception extracts the front vehicle, it does not extract the complete vehicle; In the analysis of Fig. 12c, compared with DVEM, VGG-19 focuses on the pedestrians in front, but VGG-19 also extracts the buildings and trees on the street above the picture, which has little impact on the driver's operation. Compared with the results of DVEM, Xception extracts the pedestrians in front, but Xception also extracts the redundant area at the lower left of the picture. The results show that our model can more accurately identify the driver's attention area during driving, which indicates that the prediction results of our model are reliable and sufficient to prove the superiority of the model.

**Figure 13:**
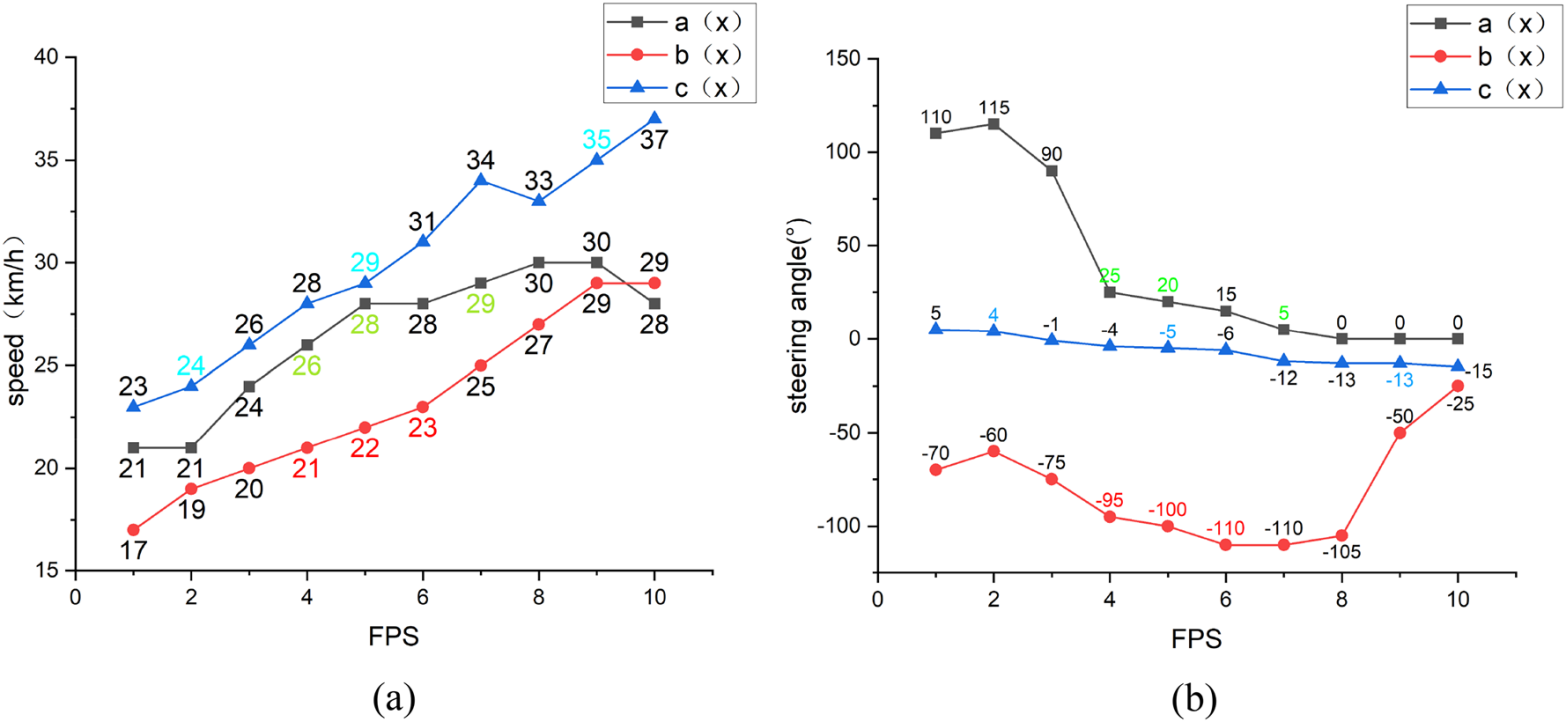
Vehicle driving state data map, where (**a**) is the vehicle speed data in three scenarios; (**b**) is the steering wheel angle data in three scenarios, a(x), b(x), c(x)in the two images corresponding to the three driving scenes of a, b, and c in Fig. 14 respectively. The three points with different colors on each broken line correspond to the three frames of images selected in Fig. 14.

**Figure 14:**
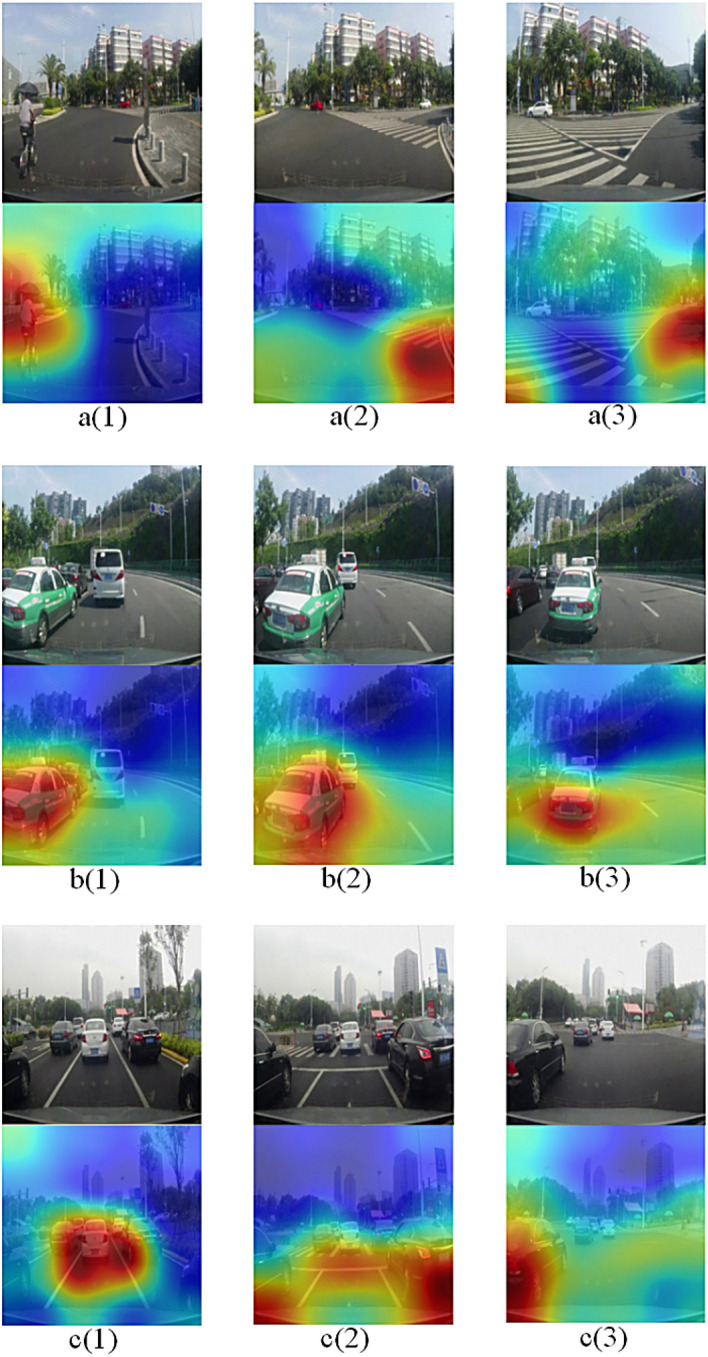
Imaging of the driver's attention area in multiple scenarios, where **a(1)**–**a(3)** the current vehicle is turning; **b(1)**–**b(3)** the vehicle in front changes lanes; there are many surrounding vehicles in the scenes of **c(1)**–**c(3)**.

Of course, the real driving scene is complicated. We select several types of typical driving scenes and combine the vehicle driving state data to verify the visualization results of the attention area. Figure 13 shows the driving state data of the vehicle when the human driver operates in the three environments. In Fig. 14, the three driving environments of turning (a), merging (b), and dense scene (c) are respectively visualized for the driver's attention area. In Fig. 13, the vehicle driving state data of 10 consecutive frames of images are selected, and the three images in each scene in Fig. 14 are included in these ten frames. The vehicle driving state data corresponding to the images selected in Fig. 14 are represented by data displayed in green, red, and blue in Fig. 13, respectively. The vehicle driving state data here is the actual vehicle data collected in the DBNet dataset.

We conduct a comprehensive analysis of Figs. 13 and 14. Combining the group "a" images in Fig. 14 and the broken line a(x) in Fig. 13b, it can be found that because there are pedestrians on the left front of the vehicle, the steering wheel angle was originally turned to the right by large amplitude, reaching + 90° in the previous frame. When the driver notices that after reaching a safe distance from the pedestrian, the steering wheel angle becomes + 25°, and at the same time, the road turns right, and the road is open. Finally, the steering wheel keeps driving at 0°. In this process, the broken line a(x) in Fig. 13a also shows that the speed of the vehicle in the open road on the right turn has risen from 26 to 30 km/h. So at this time, the driver's attention area focuses on the right turning road. The visualized images in the group "a" in Fig. 14 also shows that the attention area of the driver gradually shifts from the pedestrian on the left side of a(1) to the right area of a(3). Finally, transfer the attention area to the right turn road that is about to enter.

The broken line b(x) in Fig. 13b shows that the steering wheel is turns − 95° and the vehicle is turning left. The images in group b in Fig. 14 also show a vehicle in the front left and starting to merge. According to the broken line of b(x) in Fig. 13a, it is observed that the speed of the vehicle after the merging is 23–29 km/h, which is significantly higher than the speed change of 21–23 km/h during the merging. At this time, in the images in group b in Fig. 14, it can be seen that the attention area is the vehicle that merges into the left front, and the attention area has not left. It means that the merged vehicle affects the driver's throttle control, so the driver’s attention area is related to the merged vehicle.

The steering wheel angle is shown in c(x) in Fig. 13b is only for fine-tuning. Corresponding to the images in group c in Fig. 14, there are vehicles in front and on the left and right sides. The driver always pays attention to the surrounding vehicles. After the distance of the vehicle in front becomes farther, the amplification of speed that the collected vehicle becomes larger, so the driver's attention area is related to the vehicle in front. The attention area visualized in Fig. 14 c(3) is on the left vehicle, and the actual steering wheel angle remains unchanged at − 13° at this time on the polyline c(x) in Fig. 13b. It means that the driver noticed that the vehicle on the left did not continue to turn the steering wheel to the left to avoid a collision.

## Conclusion

Our method uses a deep neural network model to build a virtual driver model, combines the visual field image data and vehicle driving state data (steering wheel angle and vehicle speed) collected by the driver while driving through the visualization of the deep neural network. When the deep neural network virtual driver performs the same driving behavior as the actual driver, the key areas that the deep neural network model pays attention to are displayed on the driver's field of vision image and further inferred the real driver's visual attention area. The DVEM based on the residual structure is used to extract the feature of the image information, and the VSEM is used to extract the feature of the vehicle driving state data based on the time series. After fusion in the AM, the imaging result of the driver's attention area is generated.

From the results in Fig. 10, it can be found that when there is a single target in the field of view images of consecutive frames, the attention area visualized by our method is smaller, the target positioning is more accurate, and the continuous target tracking is stable. And after other targets appear, the attention area map tends to shift to other targets. In the c group part of Fig. 14, the vehicle's surrounding environment is complex, and multiple targets appear, which can also produce an attention area map. However, compared with the single target in Fig. 10, the attention area imaged by multiple targets is wider and irregular, combined with human driver visual observation habit analysis, focus on a single target, and give different levels of attention to different targets when there are multiple targets. At the same time, combined with the driver's attention area map, it can be seen that under normal driving conditions, the driver's field of vision generally pays more attention to the front and close targets.

The experimental analysis shows that the driver's attention area is closely related to the vehicle driving state data. Our method, through the imaging effect, shows the intermediate process of the neural network output results. The driver's attention area when driving is inferred using known driver's visual field image data and vehicle driving state data. This also provides help for the investigation of traffic accidents, driving behavior analysis, and intelligent vehicle assisted driving. Of course, our method still needs further improvement. We did not further classify the attention area according to the degree of attention. At the same time, further narrow the positioning range of the attention area, which is also a direction worth exploring.

## Data Availability

The data used to support the findings of this study are included within the article. All methods were performed in accordance with relevant guidelines and regulations. The dataset analysed during the current study is available at http://www.dbehavior.net/. Login code: 2277486541@qq.com; Password: yk022963.
